# Cell death as a trigger for morphogenesis

**DOI:** 10.1371/journal.pone.0191089

**Published:** 2018-03-22

**Authors:** Boris Aguilar, Ahmadreza Ghaffarizadeh, Christopher D. Johnson, Gregory J. Podgorski, Ilya Shmulevich, Nicholas S. Flann

**Affiliations:** 1 Institute for Systems Biology, Seattle, WA, United States of America; 2 Computer Science Department, Utah State University, Logan, UT, United States of America; 3 Biology Department, Utah State University, Logan, UT, United States of America; 4 Center for Integrated BioSystems, Utah State University, Logan, UT, United States of America; 5 Synthetic Biomanufacturing Institute, Logan, UT, United States of America; Queensland University of Technology, AUSTRALIA

## Abstract

The complex morphologies observed in many biofilms play a critical role in the survival of these microbial communities. Recently, the formation of wrinkles has been the focus of many studies aimed at finding fundamental information on morphogenesis during development. While the underlying genetic mechanisms of wrinkling are not well-understood, recent discoveries have led to the counterintuitive idea that wrinkle formation is triggered by localized cell death. This work examines the hypothesis that the material properties of a biofilm both power and control wrinkle formation within biofilms in response to localized cell death. Using an agent-based model and a high-performance platform (*Biocellion*), we built a model that qualitatively reproduced wrinkle formation in biofilms due to cell death. Through the use of computational simulations, we determined important relationships between cellular level mechanical interactions and changes in colony morphology. These simulations were also used to identify significant cellular interactions that are required for wrinkle formation. These results are a first step towards more comprehensive models that, in combination with experimental observations, will improve our understanding of the morphological development of bacterial biofilms.

## Introduction

Bacteria live in almost every environment. While they are critical drivers of biogeochemical cycles and ecosystem dynamics, some bacteria are major threats to human health [1–3]. Bacterial cells can attach to a surface and form a multicellular aggregate, referred to as a biofilm, which increases their survival [1]. Biofilms protect bacteria from attack by the immune system and by antibiotics and are responsible for many infections caused by implanted medical devices [4]. One of the main reasons survival is improved in biofilms is due to their complex morphologies. How bacterial assemblies develop complex morphologies has been a question pursued by many scientists. Many researchers have recently focused on the formation of wrinkles in bacterial colonies because the analysis of wrinkle formation provides fundamental information on how structural patterns can develop [5–10].

**Fig 1:**
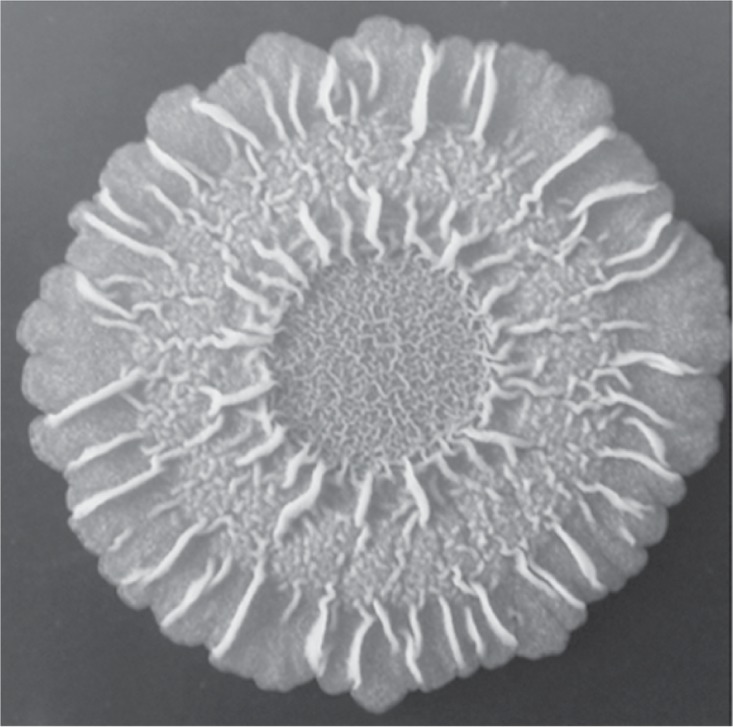
Topside of a *Bacillus subtilis* colony showing a complex interlocking wrinkled pattern. Figure adapted from Jers et al. [20].

Many bacterial colonies have a complex morphology characterized by an elaborate organization of connecting wrinkles (see Fig 1). It has been shown that these wrinkles participate in liquid transport within the colony by forming permeable channels connected in a radial network [11]. The liquid-filled channels can carry nutrients, waste, and signaling molecules. Importantly, in some bacterial colonies, such as those formed by *Vibrio cholerae* [12], the presence or absence of wrinkles distinguishes between virulent and benign states.

Wrinkles can form in tissues through mechanical instabilities that are generated by constrained growth of tissues with specific elastic properties [13–16]. This physical mechanism has been suggested for the development of many wrinkled or undulated morphologies, such as the wrinkled morphology of the brain, tubular organs, and some biofilms [17–19]. A novel mechanism of wrinkle formation was revealed in a recent study by Asally et al. [9] which showed that localized cell death initiates wrinkle formation in *Bacillus subtilis* colonies. The abundant extracellular polymeric substance (EPS) produced by cells plays a critical role in wrinkle formation, underlying the formation of local regions of cell death and providing a mechanical support that resists compressive forces stemming from cell displacement driven by cell growth and cell division. Cell death disrupts the integrated network of cells and EPS within the biofilm, providing an outlet for compressive stress [9].

Complexly organized biofilms start from a single bacterium adhering to a surface. The bacterium secretes a glue-like protein that attaches it more tightly to the substratum. Upon division, the daughter cells are cemented together and to the substratum [8]. These cell-cell and cell-surface bonds, coupled with the pressure arising from population growth, push the expanding colony into a quasi-stable state in which unrelaxed forces are dampened by the rigid structure of biofilm. This rigid structure is formed by the EPS that wraps around the cells and provides the biofilm both mechanical support and resilience against environmental stresses [21–24]. Significantly, EPS production is essential for biofilm wrinkling [22, 25, 26].

A quasi-stable state is reached between 24 to 48 hours of biofilm development when the colony appears as a smooth, disk-like structure [9]. Continued growth leads to the formation of an intricate colony-wide pattern of cell death at the colony-substratum boundary in response to nutrient depletion, high cell density, and waste accumulation (see Fig 1 in Asally et al. [9]). In regions of cell death, the colony detaches from both, the substratum and surrounding cells, and the biomass converges to the areas opened by the dying cells. This leads to buckling of the colony into a complex pattern of interlocking wrinkles illustrated in Fig 1.

The focus of this study is on the transition from a smooth, stiff colony under compression to a complex wrinkled morphology triggered by localized cell death. This study does not consider the development of the smooth compressed colony or model how cell death patterns emerge, but begins with the initiation of realistic patterns of cell death at the colony-substratum interface.

We developed an agent-based model that considers cells and associated EPS as single agents to study the formation of 3D cellular structures that result from the interplay of cell death and biomechanical forces, and implemented this model using the *Biocellion* simulation framework [27]. Agent-based modeling is becoming a popular modeling framework to investigate the influence of mechanical properties on biological systems, including biofilms [28–32]. Agent-based approaches allow the integration of inter- and intra-cellular interactions and the exploration of cellular heterogeneity [33]. Our aims were to: a) test the hypothesis that cellular mechanical approaches allow the integration of intracellular interactions, b) explore how cellular interactions can both power and control wrinkle formation in biofilms in response to localized cell death, c) to learn how changes in mechanical properties of biofilms affect the structure of wrinkles, and d) to identify the intercellular interactions needed to form wrinkles.

## Model description

**Fig 2:**
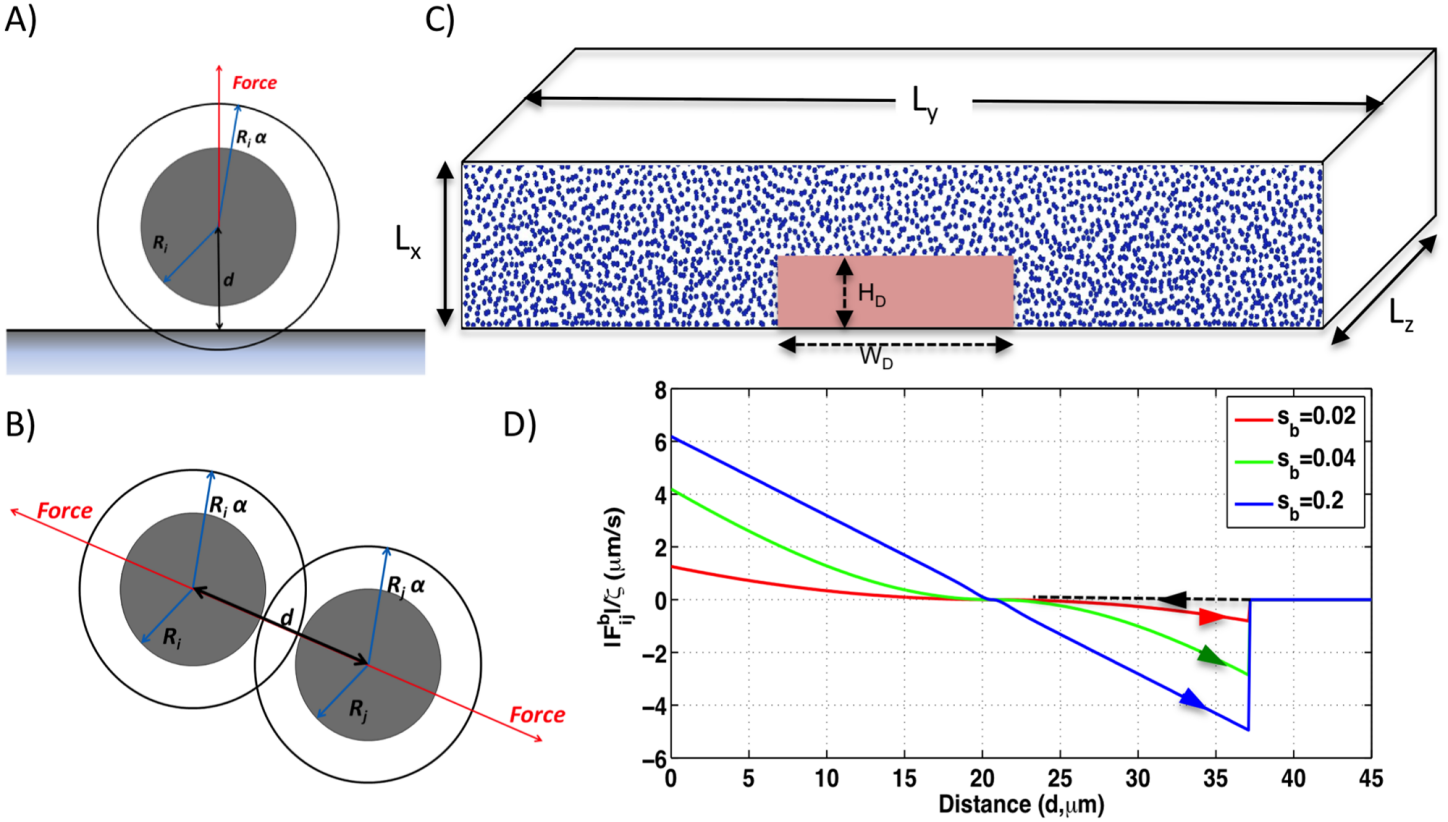
Model specifics. Cartoon representation of cell/boundary (A) and cell/cell (B) elastic forces. *R*_*i*_ is the radius of cell *i*, *α* is the EPS shoving factor and *d* is the distance between the objects. (C) A representation of the initial configuration of cells and the parameters that define the size of the simulation system and the cell death pattern (Red). (D) Forces between two cells based on their distance for different values of *s*_*b*_(colors). The dashed lines indicate that the mechanical force for nonbonded cells is zero.

To study mechanical effects on wrinkle formation, we developed an agent-based three dimensional model. Each agent is a sphere that represents a bacterial cell and a small amount EPS attached to the cell surface. The radius of an agent is *αR*_*i*_, where *R*_*i*_ is the radius of a bacterial cell, and *α* is a scaling factor (*α* > 1) that accounts for the space taken up by the mass of EPS that is attached to the cell wall (Fig 2B). Agents move in an overdamped environment in which viscous forces a have stronger effect than inertial forces. We used the equations of Brownian dynamics (BD) to model the dynamics of agents [33]. The velocity of an agent is the sum of two components: a term that is proportional to the net force acting on the agent [28], and a random term. In our implementation, the BD equations are integrated with the discrete time Euler-Maruyama scheme, so that the position **x**_*i*_ of every cell *i* changes at every time step Δ*t* according to:

$$\begin{array}{c} x_{i}\left(t+\Delta t\right)=x_{i}\left(t\right)+\Delta t\;f_{i}^{a}\left(x\right)/\zeta +\sqrt{2D_{c}\Delta t}\;\xi \left(t\right) \end{array}$$ (1)

where *ζ* is the coefficient of viscous friction, *D*_*c*_ is the diffusion coefficient of agents, and ξ(*t*) is a vector with components that are random numbers sampled from a normal distribution with zero mean and unit variance. $f_{i}^{a}\left(x\right)$ is the sum of all mechanical forces that act upon agent *i*, and depends on the current positions of the agents (**x**). We implemented two simple mechanical forces: those between pairs of agents and the forces between agents and the agar substratum:

$$\begin{array}{c} f_{i}^{a}=\sum_{j}^{}f_{ij}^{b}+f_{i}^{ba} \end{array}$$ (2)

The sum on the right hand side considers all agents *j* that are bonded to *i*. A bond is created between two agents when the distance between their centers becomes smaller than a threshold value *δ*_*c*_. Similarly, a bond between two agents is broken when the distance between their centers becomes larger than *δ*_*d*_. The force between bonded agents is described by the following equations:

$$\begin{array}{ccc} x_{ij} & = & \alpha R_{i}+\alpha R_{j}-d \end{array}$$ (3)

$$\begin{array}{ccc} \left|f_{ij}^{b}\right| & = & Kx_{ij}\tanh\left(s_{b}|x_{ij}|\right) \end{array}$$ (4)

where *d* is the distance between the centers of agents *i* and *j*. The bond between two agents, with the parameter *K* being the spring constant of the bonds, is an attractive force when the distance is greater than *α*(*R*_*i*_ + *R*_*j*_) and a repulsive force when the distance is less than *α*(*R*_*i*_ + *R*_*j*_). The attractive force between a pair of agents grows with distance until the bond breaks and the agents become unassociated. The stiffness of the bond between two agents is controlled by the parameter *s*_*b*_. As *s*_*b*_ increases, the forces around the equilibrium point also increase to pull or push the cells back to the equilibrium point, more strongly enforcing the distance constraint (Fig 2D). With small *s*_*b*_, the distance constraint is lax and the agents are allowed to separate away from the equilibrium distance even when weak forces are applied (Fig 2D). The force between agents and the agar substrate is modeled similarly. The magnitude of the force is $|f_{i}^{ba}|=Kx_{ii}\tanh\left(s_{ba}|x_{ii}|\right)$, where *x*_*ii*_ = *αR*_*i*_ − *d*. In this case, *d* is the distance of the center of agent *i* to the agar surface (Fig 2A). The direction of the force is perpendicular to the horizontal agar surface. A bond between a cell and agar surface is created if *d* is smaller than a threshold value *δ*_*ca*_, and the bond is broken if *d* becomes greater than *δ*_*ca*_.

The initial configuration of the simulations represents the stiff colony before the onset of cell death and subsequent wrinkling. It consists of a set of agents randomly located in a rectangular box (Fig 2C). The initial positions of the cells filling the box with a specific value of volumetric density (*Φ*) are generated by the program SmolCrowd [9, 34]. To generate initial configurations of colonies in a compressed state, the values of *Φ* are selected such that average distance between agents is smaller than the rest length (2*αR*_*i*_). A subsequent small simulation with weak forces is applied to homogenize the arrangement of cells (see S1 Text in Supporting Information).

Cell death is modeled by removing the agents with centers located in the volumetric region of the cell death pattern (CDP) at the colony-substratum interface, depicted as a red rectangle in Fig 2C. In the following sections we will refer to agents that are composed of cells and local EPS simply as cells.

## Results

### Correlation of cell death and wrinkle location

**Fig 3:**
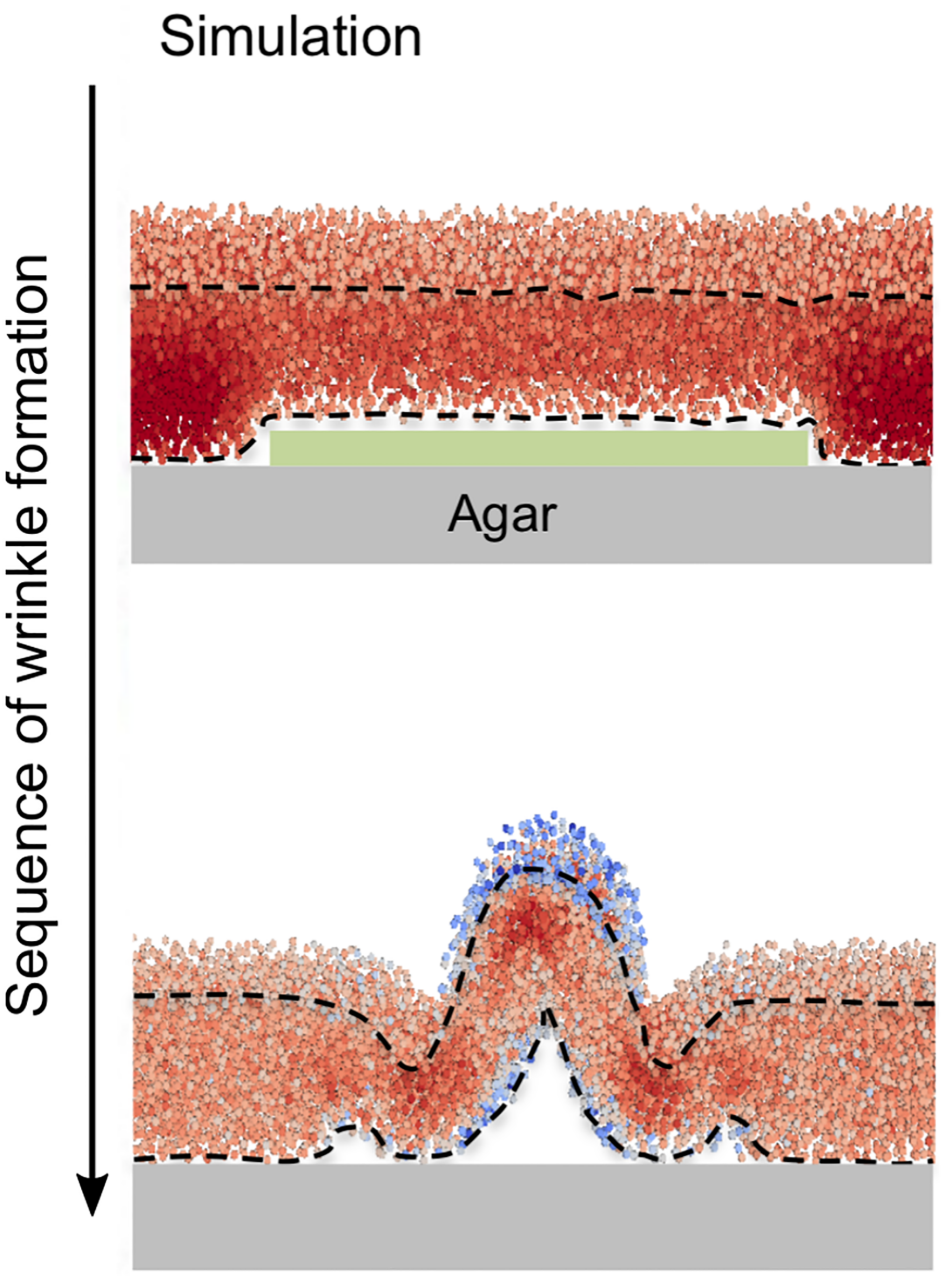
Sequence of wrinkle formation due to cell death at the cell-agar interface. The figure shows a cross-sectional image of simulated wrinkle formation. Green indicates the area of cell death. Cells are colored by according to the mechanical stress they experience (red, compression; blue, tension). Please refer to Fig 4G of Asally et al. [9] for a comparison with wrinkle formation observed in a *B. subtilis* colony.

To explore the hypothesis that the biomechanical forces unleashed by cell death are sufficient for forming wrinkles in biofilms, we simulated the morphogenesis of colonies that were initially configured in a compressed state that simulates cells fully encased in EPS. The mechanical support provided by the EPS is modeled by elastic bonds between adjacent cells. To model the effect of cell death, the network of bonds at the interface of the colony and the agar substratum is broken by removing cells from the system. As Fig 3 shows, the subsequent converging movements of the cells cause vertical buckling where cell death occurred. Fig 3 demonstrates our 3D simulation of this process where cell death (the green rectangle) disrupts the connection between the biofilm and the agar substratum, and perturbs the distribution of mechanical stress. The results of this simulation are in good agreement with *in vivo* observations that regions of cell death that occur at the interface between a bacterial colony and the agar medium presage areas where the colony later buckles (see Fig 4G of Asally et al. [9]). Spheres in Fig 3 represent cells that are colored according to pressure (red, for compression; blue, tension; see Methods for details about the computation of pressure). Cell death creates a heterogeneous distribution of compressive stress. A region of low stress appears at the top of the region of cell death and this induces convergent horizontal cell movement towards the center of the cell death region.

**Fig 4:**
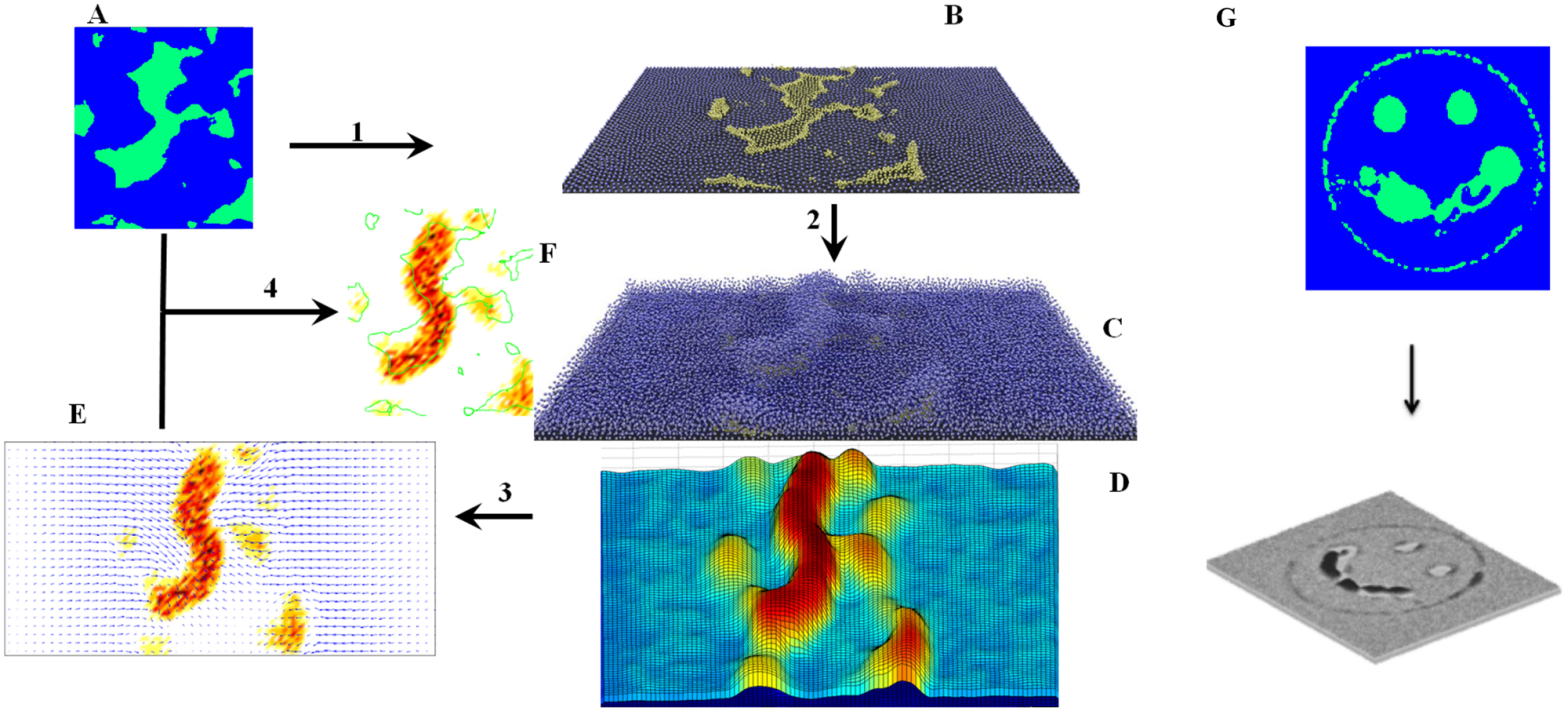
Outline of the study. A: Cell death pattern (CDP). B: CDP mapped to the bottom layer of a colony in which the cells are in a quasi stable state. The upper layer of the colony is not shown in this image. C: Relief of lateral mechanical stress at the CDP area gives rise to the wrinkles. D: Reconstructed surface of biofilm. E: Velocity vectors and convergence of vector fields computed from material movement. F: Spatial correlation of CDP (green) and wrinkles (red). G: “Smiley face” simulation: an artificially designed cell death pattern results in wrinkle formation in the simulation shown in the lower panel (Grey scale proportional to height).

To track the movement of particles during wrinkle formation, we simulated the experimental setup of Asally et al. [9]. Fig 4 outlines the computational experiment. Starting with a block of cells under mechanical stress, we mapped the cell death pattern (CDP), as shown in Fig 4A, to the bottom layer of this block, then ablated the cells in the mapped area (Fig 4B). As expected, wrinkles form above the areas of cell death as shown in Fig 4C. We discretized the surface by overlaying a grid on the simulation domain and summing the trajectories of the particles in each square of the grid to compute the velocity vector for that partition (blue arrows in Fig 4E). These velocity vectors determine the convergence (negative divergence) of vector fields, demonstrating the aggregate material directional movement. The colored areas in Fig 4E show the convergence, where a more intense color corresponds to higher convergence, (see S1 Fig in Supporting Information for a larger figure). The observed convergence along with the velocity vectors confirm the idea that wrinkles can be formed by local cell death [8]. Fig 4F shows the spatial correlation between areas of cell death and wrinkles. Fig 4G demonstrates a more complex simulation that reproduces a “smiley face” that arises from a CDP similar to the one shown in Fig 4H of Asally et al. [9]. When the designed cell death pattern is incorporated in the simulations, a smiley face of wrinkles appears on top of the biofilm (bottom panel of Fig 4G).

### Mechanical stiffness influences wrinkle height and width

Studies in material science have shown that geometrical features of wrinkles in thin films are greatly affected by the mechanical properties of the film [35, 36]. To investigate this effect in biofilms, we simulated three strains of *Bacillus subtilis* (wild type (WT), *δarfB*, and *δsrfA*) reported to form colonies with different degrees of stiffness due to the amount and composition of EPS that they produce [9]. Compared to wild type *B. subtilis*, *δarfB* generates a softer biofilm in which EPS production is repressed [37]. In contrast, *δsrfA* generates a stiffer biofilm in which EPS production is increased [38]. Assally et al. [9] observed that colony stiffness correlates with wrinkle size and that *δsrfA* strains have larger wrinkles than WT or *δarfB*. We tested whether our modeling approach could reproduce this observation.

**Fig 5:**
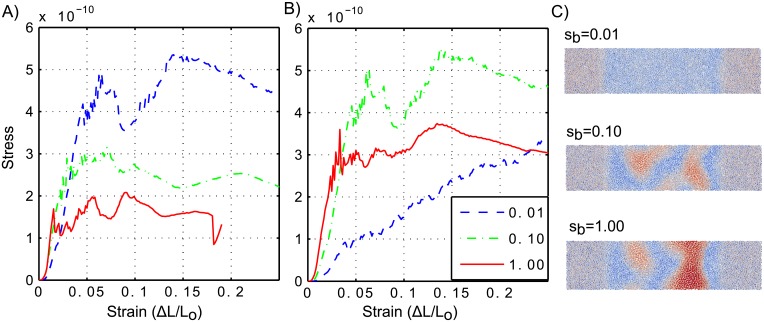
Stress-strain simulation experiments. We used three values of bond flexibility (*s*_*b*_ values in color) and two values of *K* (*K* = 20.0 in panel **A**, and *K* = 2.0 in panel **B**). **C**: Top view of the colonies at the end of the simulation of colonies perturbed by cell death, for *K* = 2.0. Color indicates the height, red for higher values and blue for lower values. For this simulation, we used *W*_*D*_ = 1000 and the parameter values specified in S1 Table of the Supporting information.

In order to determine the mechanical stiffness of our simulated colonies, we performed uniaxial compression experiments, similar to the computational experiments described in Pathamatan et al. [39]. The simulation domain for these experiments was a rectangular region with *L*_*x*_ = 90, and *L*_*y*_ = *L*_*z*_ = 360*μm*. The agents were randomly located in the rectangular region with a volumetric density of 0.6. Before the compression experiments, the agents were subjected to an homogenization stage in which the system is simulated for 2000 steps to equilibrate the inter-cellular forces (see S1 Text of the Supporting Information for details of homogenization stage). The compression experiments were performed with periodic boundary conditions in the *x* direction, an impenetrable wall at *y* = 0; and no restriction on the *z* direction. The cells located at the border (those that overlap with the plane *y* = *L*_*y*_) are displaced a small distance, Δ*L*, in the −*y* direction. The system is then allowed to evolve according to the equations of motion (Eq 1). During the simulation the *y* coordinates of the border cells are fixed. The biofilm segment is simulated until *v*_*max*_ < 0.01*μms*^−1^, where *v*_*max*_ is the maximum velocity of the cells. At the end of the simulation, the force on the compressed surface is computed as the sum of the forces in the *y* direction of all border cells. Stress is then estimated by dividing the average force by the number of cells located at the border. The experiment is repeated with cells located at the border subjected to an additional displacement Δ*L*. Fig 5A and 5B show the stress-strain curves obtained by the computational experiments for different parameters that modulate colony stiffness. Stiffness is characterized by the slope of the curve at the initial (linear) part of the stress-strain experiments; stiffness increases with the slope of the stress-strain curve.

Within our modeling framework, there are two parameters that can potentially modulate the colony stiffness: bond flexibility (*s*_*b*_) and the spring constant (*K*). Fig 5A and 5B shows the stress-strain curves of the compression experiment for two values of *K* (20.0 and 2.0) and three values of *s*_*b*_ (1.00, 0.1, and 0.01). Fig 5C shows a top view at the end of simulated wrinkle formation due to cell death for each of the three values of *s*_*b*_ and *K* = 2.0. The plots show that, for a constant value of *K*, *s*_*b*_ determines colony stiffness, i.e. the slope of the stress-strain curve in the linear regime. For a high value of *K* (Fig 5A), even though the colonies have different stiffnesses, they behave similarly. An initial linear regime is followed by a plastic region in which the stress becomes almost constant, a trend that characterizes a material that has the capacity to store energy due to compression. For a low value of *K* (Fig 5B), we observe two distinct material behaviors. Colonies with *s*_*b*_ = 1.00 and *s*_*b*_ = 0.10 behave similarly to those shown in Fig 5A. A distinct behavior is observed for *s*_*b*_ = 0.01 in which the stress response constantly increases with strain. Notably, this is the only case in which no wrinkle is formed when there is cell death (Fig 5C). Simulations of wrinkle formation due to cell death show that colony stiffness affects the morphology of wrinkles. Stiffer colonies (with higher *s*_*b*_) form wider and higher wrinkles (see Fig 5C). This is in good agreement with experiments performed with mutant strains that form colonies with lower and higher stiffness than wild type.

**Fig 6:**
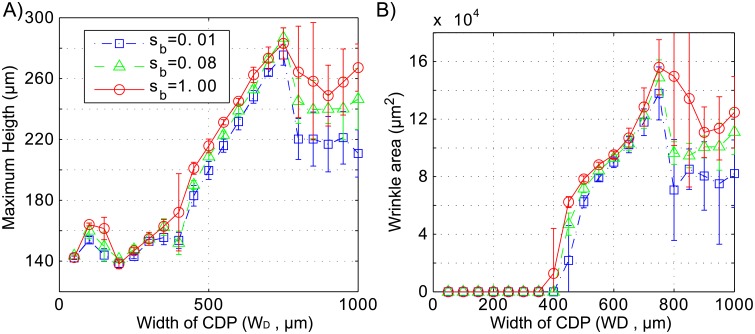
Effect of colony stiffness on wrinkle size. **A.** Colony height versus the width of the cell death region (*W*_*D*_) for three values of colony stiffness (*s*_*b*_). **B.** Wrinkle area versus *W*_*D*_ for three values of *s*_*b*_. Every point represents the average of 6 simulations with random initial positions and the bars indicate standard deviations.

To analyze in more detail how the colony stiffness affects the morphology of wrinkles, we performed simulations of wrinkle formation induced by cell death for *s*_*b*_ = 0.01, 0.08, and 1.00, Φ = 0.16, and a set of cell death widths (*W*_*D*_ = 50, 100, …, 1000). All other parameters were set according to S1 Table of the Supporting Information. For each pair of *W*_*D*_ and *s*_*b*_, we performed 6 simulations corresponding to different initial distributions of cells. The relationships between cell death area (*W*_*D*_) and wrinkle height and area are shown in Fig 6A and 6B, respectively, for three different values of colony stiffness (*s*_*b*_). Wrinkles generated by *s*_*b*_ = 1.00 are higher than those generated by *s*_*b*_ = 0.08 and 0.01, for all values of *W*_*D*_ except *W*_*D*_ = 750*μm*. The difference in wrinkle height and area is more noticeable for larger areas of cells death (*W*_*D*_ > 750) in which average wrinkle height and area are always larger for greater values of colony stiffness *s*_*b*_. These results are in good agreement with experimental observations in which colonies with higher stiffness (mutant *δsrfA*) generate larger wrinkles than colonies with lower stiffness (WT and *δarfB* strain).

**Fig 7:**
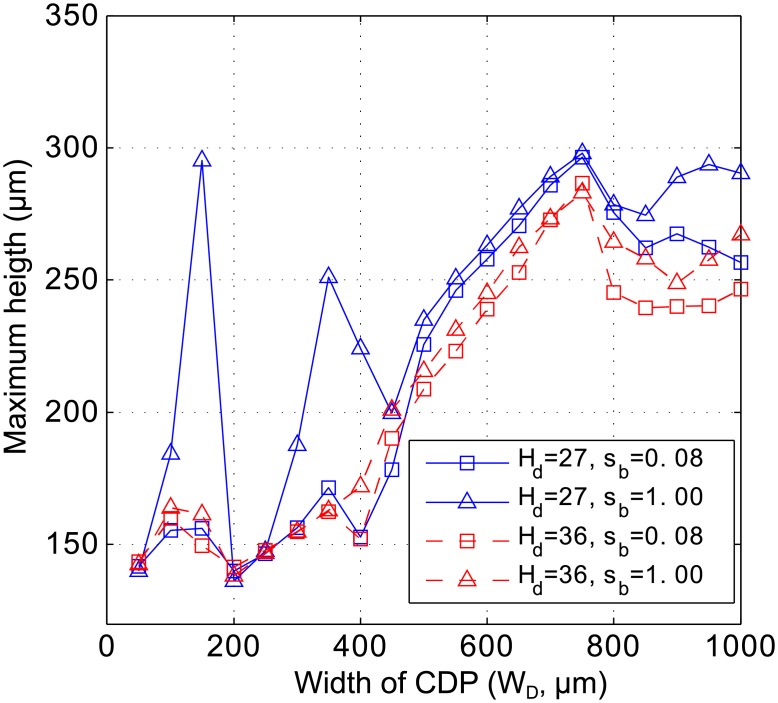
Colony height versus the width of cell death region (*W*_*D*_), for different values of the cell death region height (*H*_*D*_) and colony stiffness (*s*_*b*_).

**Fig 8:**
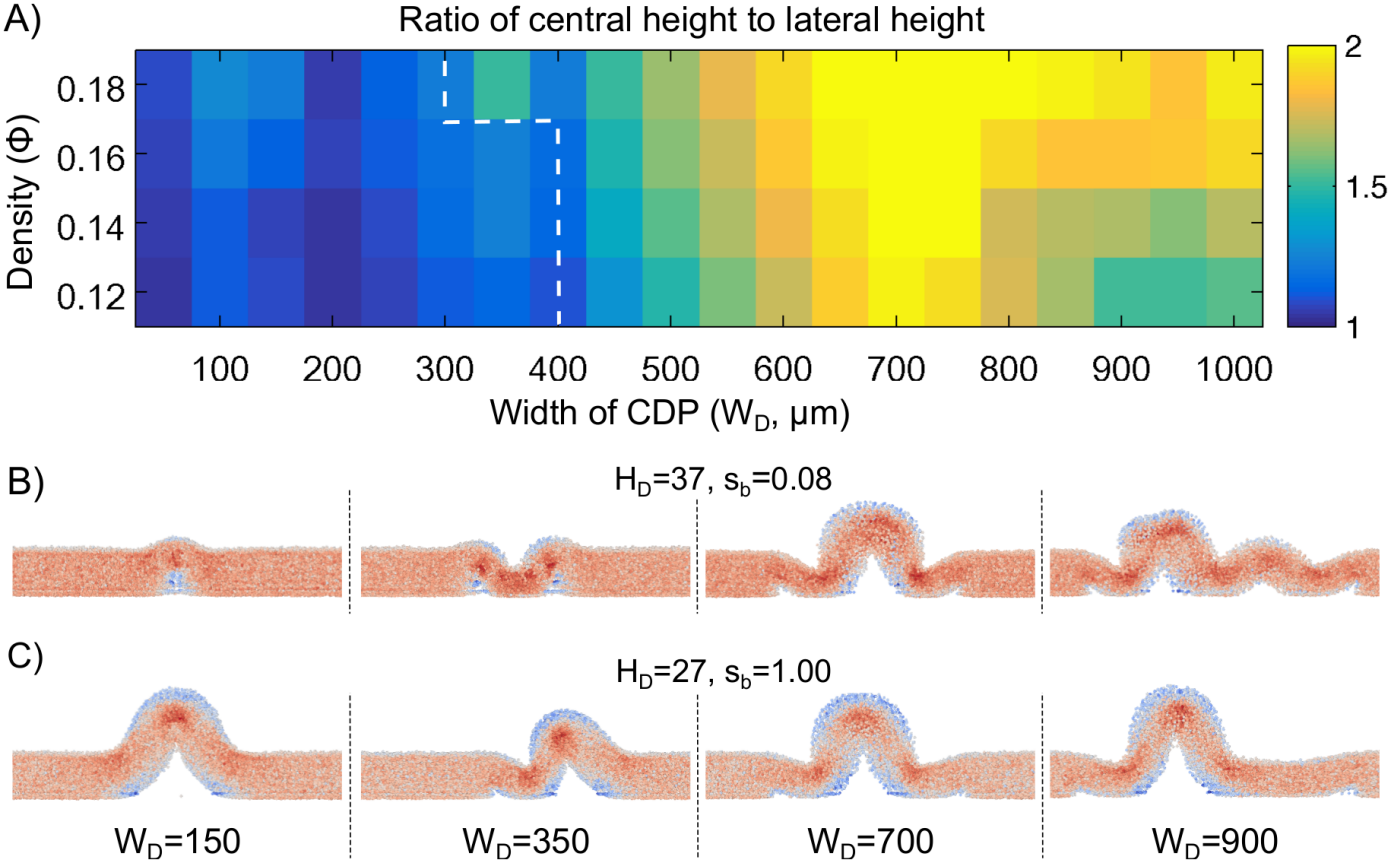
Effect of volumetric density and cell death size on wrinkle formation. A: Ratio of central to lateral height of simulated colonies for several values of initial volumetric density (Φ) and width (*W*_*D*_) of the cell death pattern (CDP). The white dashed lines highlight a region in which cell death does not induce wrinkle formation. B: Representative side views at the end of simulations for different values of *W*_*D*_, *s*_*b*_ and *H*_*D*_. The color intensities represent computed mechanical stress (see Fig 3). *W*_*D*_ units are in micrometers.

In Fig 6A, we observe three local maxima of wrinkle height which are associated with different wrinkle morphologies. Higher wrinkles and different morphologies are observed for smaller heights of the cell death region (smaller values of *H*_*D*_), which produce smaller volumes of cell death. Fig 7 compares wrinkle heights obtained from different values of *H*_*D*_ and colony stiffness. For *H*_*D*_ = 27*μm* and *s*_*b*_ = 1.00 there are three wrinkle height local maxima. A tall wrinkle is formed at *W*_*D*_ = 150. This wrinkle almost disappears if *H*_*D*_ is increased to 36 or if *s*_*b*_ is decreased to 0.08. Wrinkle height also peaks at *W*_*D*_ = 350 and 750. The morphology of these wrinkles is shown in Fig 8C. This pattern resembles the morphology observed in side views of wrinkles of bacterial colonies (see Fig 4G of Asally et al. [9]). Fig 7 also shows that reducing the volume of the cell death region by changing *H*_*D*_ from 36 to 27 *μm* generates higher wrinkles. This non-intuitive observation may be explained by considering that a smaller volume of cell death leaves a greater biomass in the biofilm to be accommodated after cell death.

### Determinants of wrinkle formation by parameter exploration

One of our goals is to use modeling to identify parameters that are critical for biofilm morphogenesis that can be later tested in experimental settings. Here, we used the model to determine the sensitivity of wrinkle formation to different parameters. Our strategy was to systematically change the value of one or two parameters in the simulation while keeping the other parameters constant. These simulations were performed in a rectangular group of cells with varying regions of cell death located at the center of the simulation domain (Fig 2C).

First we explored a range of cell death widths (*W*_*D*_ = 50, 100, 150, …, 1000) and volumetric densities (Φ = 0.12, 0.14, 0.16, 0.18) to identify parametric regions that allow colonies to form wrinkles. For each combination of *W*_*D*_ and Φ, a set of 3 simulations, corresponding to different initial positions of cells, was performed with stiffness parameters *s*_*b*_ = *s*_*ba*_ = 0.08, *D*_*c*_ = 0 (no cellular motility), and the additional parameters specified in S1 Table of the Supporting material. Fig 8A shows a heatmap of the ratio of height at the center of a colony to the height at the border of a colony (central height to lateral height ratio in Fig 8A, *r*_*h*_) at the end of the simulations. The procedure used to compute the height of the colony at the border (*y* = 0) and the center (*y* = *L*_*y*_/2) is described in the Methods section. A value of *r*_*h*_ = 1 (blue) indicates that the height of the center and the border are equal, i.e., no wrinkle had formed. Values close to 2 (yellow) indicate that one or two wrinkles had formed in the center of the colony. The lower left part of the heatmap of Fig 8A shows a parametric region of *W*_*D*_ and Φ in which no wrinkles form. These results suggest that for relatively small volumetric densities, wrinkles do not form if the region of cell death is small. We visually inspected the morphologies at the end of the simulations used to obtain the heatmap of Fig 8; representative side views are shown in Fig 8B. These snapshots suggest that the size of the cell death region determines the final morphology of the wrinkles. Specifically, the number of wrinkles that form at the top of the cell death region depends on its width. This observation is in good agreement with observations of bacterial colonies in which larger regions of cell death generate two parallel wrinkles while relatively small regions of cell death generate a single wrinkle [9].

**Fig 9:**
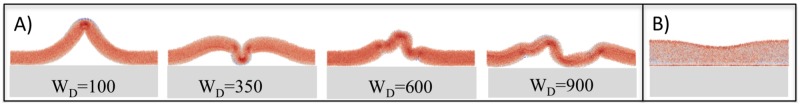
Side views of colonies obtained at the end of simulations with adhesive interactions turned off. A: Final morphologies from simulation in which only the adhesive interactions between cells and the agar surface are turned off. B: Final morphology (*W*_*D*_ = 600) obtained from simulations in which cell-cell adhesive interactions are turned off. In all cases in panels A and B, a volumetric density of Φ = 0.16 was used.

The effect of the adhesive forces on colony morphology was investigated by individually turning off agent-agent and agent-substratum adhesion. First, we turned off only adhesive interactions between agents and the agar surface, i.e., *s*_*ba*_ = 0 when the distance between the center of a cell and the agar surface is larger than *αR*_*i*_. The remaining parameters were at the values specified in the first part of this section. Fig 9A shows side views of colonies at the end of the simulations for different sizes of the cell death region and Φ = 0.16; other values of Φ generate similar morphologies. These results suggest that a loss of cell agar surface adhesion does not block the formation of wrinkles. However, surface adhesion does have a great impact on colony morphology as observed in Fig 9A. A loss of adhesive bonds to the agar surface reduces stability, allowing cells to reaccommodate in ways that generate morphologies substantially different from those of actual bacterial colonies. We also turned off the adhesive interaction between cells, i.e. *s*_*b*_ = 0 when the distance between two cells is larger than *α*(*R*_*i*_ + *R*_*j*_). The remaining parameters were fixed identically to the previous experiment. Fig 9B shows a side view of the colony at the end of the simulations for *W*_*D*_ = 600, other values of *W*_*D*_ produced similar morphologies. In agreement with previous observations [9], these results confirm that cell-cell adhesion is essential for the formation of wrinkled morphologies.

## Discussion

To form a multicellular aggregate, such as a biofilm, cells interact via a complex interplay between biochemical signaling and biomechanical forces. However, these interactions are still poorly understood. Investigating the morphogenesis of model biological systems, such as biofilms, is important for understanding and formalizing the common patterns seen in more complex systems and organisms. Recent studies showed that cell death triggered by biochemical stress combined with a relaxation phase of biomechanical stress, plays a critical role in the initiation of wrinkles in biofilms.

We developed an agent-based model to evaluate the effect of cellular level mechanical interactions on wrinkle formation due to cell death. We modeled mechanical interactions through the implementation of elastic bonds between pairs of agents and also between agents and the agar surface. In this model, an agent is a cell and the surrounding EPS. Cell death was modeled by removing agents from the system. Because instantaneous deletion of cells may be a more drastic perturbation than encountered in biological systems, we have also performed simulations in which cells are gradually removed from the system. We found that abrupt or gradual removal of cells produced the same final results (see S1 Text in Supporting Information). By implementing the cellular processes of mechanical interactions and cell death, we were able to qualitatively recapitulate the process of wrinkle formation that are observed in colonies of *Bacillus subtilis* [9]. Although this simple model performed well, a more complete model would include other cellular events, such as cell density change due to cell division, cell motility, the effect of waste molecules and nutrients, and heterogeneity of EPS production.

We first aimed to investigate the role of bond stiffness on wrinkle morphology. We found that bond stiffness is the major modulator of colony stiffness. By changing the bond stiffness, we simulated colonies with distinct mechanical behaviors. Colonies that generate wrinkles when perturbed by cell death have a mechanical behavior characterized by a linear elastic regime followed by plastic-like behavior. Colonies without plastic-like behavior did not form wrinkles after cell death. Moreover, colonies simulated with higher stiffness generated wider and higher wrinkles, in good agreement with observations of bacterial colonies [9]. In the simulations, the relationship between wrinkle height and stiffness is more pronounced for wider cell death regions. Furthermore, decreasing the height of the cell death region resulted in larger wrinkles, even when cell death regions are relatively small. This non-intuitive result suggests a complex interplay between the geometry and volume of the cell death region and wrinkle morphology. In actual biofilms, it is likely that the volume of cell death regions is influenced by colony stiffness, which will require a model that relates cell death to mechanical stress.

We performed simulations that correspond to different sets of parameters that characterize our model. This approach helped us identify properties beside mechanical stiffness that determine wrinkle formation. Our results suggest that small cell death regions are less likely to trigger wrinkles in colonies with low cell densities. Moreover, the size of cell death regions determines the morphology of the wrinkles; large cell death regions produce multiple wrinkles with specific wavelengths, whereas smaller cell death regions generate a single wrinkle on top of the region of cell death. Our results also show that cell-cell adhesion is essential for wrinkle formation. However, while cell adhesion to the agar substratum influences colony morphology, its suppression does not completely prevent the formation of wrinkles.

The wrinkle formation simulated in this study represents one specific morphological feature of the whole colony. Future work will include expanding the current simulation to a larger spatial scale, including other biological events such as cell division, the effects of nutrients and waste molecules, as well as intracellular gene regulatory networks that modulate the primary determinants of wrinkling, cell adhesion and cell death.

## Methods

### Agent based framework

We implemented the wrinkle formation model (see Model description) in *Biocellion*, an HPC simulation framework designed to accelerate agent-based simulations of biological systems composed of millions to billions of cells. *Biocellion*, which runs on clusters and has been tested in a cloud computing environment, is already capable of producing high-fidelity simulation data for living cell system models having billions of cells, such as yeast patterning, bacterial colonies on soil aggregate, and cell sorting [27, 40]. The code of our implementation of the model is freely available with a set of inputs that were used to simulate the process of wrinkle formation.

### Convergence computation

In vector calculus, convergence (negative divergence) is a vector operator that represents the volume density of inward flux of a vector field around a given point. As mentioned in the main text, we partition the *xy* plane into a set of mesh cells using a Cartesian mesh. For each particle we track the movement and compute the displacement vector. We compute the convergence at a particular mesh cell by subtracting the number of particles that enters that mesh cell from the number of particles that left.

### Vector field

In vector calculus, a vector field is an assignment of each point with a velocity vector. The velocity vector for each voxel is computed as the aggregation of all displacement vectors of particles which were initially located in that voxel.

### Computation domain

In simulations of wrinkle formation, we set up periodic boundary conditions in the *y* and *z* directions and rigid walls in the *x* direction. *x* = 0 represents the agar surface that supports the colony. For the simulation shown in Fig 3 we used dimensions of *L*_*x*_ = 90, *L*_*y*_ = 1440, and *L*_*z*_ = 360 micrometers and a volumetric density of Φ = 0.16. In Fig 4G we used approximately 800,000 cells structured in a 90 × 4500 × 3600 micrometer block, and a 200 × 9600 × 3500 micrometer block in the experiment of Fig 4C. The generation of cells within the computation domain with each volumetric density (Φ) is described in S1 Text of the Supporting Information.

### Computation of mechanical stresses

In our agent based model, individual cells are explicitly included, allowing the modeling of mechanical interactions. In particular, the mechanical forces in a given spatial configuration of cells provide important information about the spatial distribution of mechanical stresses in the interior of the colony. The local stress tensor at cell *i* of a colony in a given configuration is [33, 41]:

$$\begin{array}{c} \sigma^{i}=\frac{1}{V_{i}}[\frac{1}{2}\sum_{j}f^{ij}⊗r^{ij}] \end{array}$$ (5)

where *V*_*i*_ is the volume of each spherical agent, **f**^*ij*^ is the force acting on agent *i* due to agent *j*, and **r**^*ij*^ is the position of the center of cell *j* relative to the center of cell *i*. Similarly to Fenley et al. [41], we compute the hydrostatic stress for visualization of figures in the main text:

$$\begin{array}{c} \sigma_{hyd}^{i}=\text{trace}\left(\sigma^{i}\right)=\left(\sigma_{xx}^{i}+\sigma_{yy}^{i}+\sigma_{zz}^{i}\right)/3 \end{array}$$ (6)

where $\sigma_{hyd}^{i}$ is a scalar value that can be visualized for each cell. In the main text, $\sigma_{hyd}^{i}$ is referred to as the mechanical stress that a cell supports due to mechanical interactions.

### Colony height

To compute colony height (*H*), we divide the simulation domain into regular voxels of 20 × 20 × 20*μm*. Each voxel is determined by three indices (*i*, *j*, *k*), which represent its position in the *x*, *y*, and *z* directions, respectively. For every voxel we compute the average height of the cells located in that voxel:

$$\begin{array}{c} H^{\left(i,j,k\right)}=\frac{1}{N_{i,j,k}}\sum_{l}^{N_{i,j,k}}h_{l}^{\left(i,j,k\right)}, \end{array}$$ (7)

where *N*_*i*,*j*,*k*_ is the number of cells located in voxel (*i*, *j*, *k*), and $h_{l}^{\left(i,j,k\right)}$ is the height (*x* coordinate) of cell *l* located in the voxel. The height of the colony is then computed as:

$$\begin{array}{c} H=\max_{i,j,k}\left(H^{\left(i,j,k\right)}\right). \end{array}$$ (8)

The height of the border of the colony is computed as *H*^*j*=0^ = max_*i*,*k*_(*H*^(*i*,0,*k*)^), and the height of the center of the colony is *H*^*j*=72^ = max_*i*,*k*_(*H*^(*i*,72,*k*)^) for the system in which *L*_*x*_ = 1440*μm*. These values are used to compute the border to center height ratio (*r*_*h*_) used in Fig 8A.

### Wrinkle area

To estimate the wrinkle area at the end of a simulation (Fig 6B), we divide the simulation domain into regular 20 × 20 × 20*μm* voxels, and compute the maximum height in the *x* direction, *h*_*x*_(*j*, *k*) = max_*i*_ *H*(*j*, *k*). The matrix *h*_*x*_(*j*, *k*) is then mapped into a binary image using a threshold value of 1.3*H*^*j*=0^, where *H*^*j*=0^ is the height of the border of the colony. Other threshold values generated a similar trend than the one shown in Fig 6B. Finally, the wrinkle area is computed as the maximum area of the connected components of the binary image.

## Supporting information

S1 Fig Vector field and convergence for biofilm surface, demonstrating the aggregate material directional movement. (TIFF)

S1 Table Parameters, constants, and expressions used in the simulations. (PDF)

S1 Text Supplementary information for “Cell death as a trigger for morphogenesis”. (PDF)
