# Supplementary figures and images for: Cell death as a trigger for morphogenesis
Source: PLoS One. 2018 Mar 22;13(3):e0191089. doi: 10.1371/journal.pone.0191089 (PMC5863959; doi:10.1371/journal.pone.0191089)

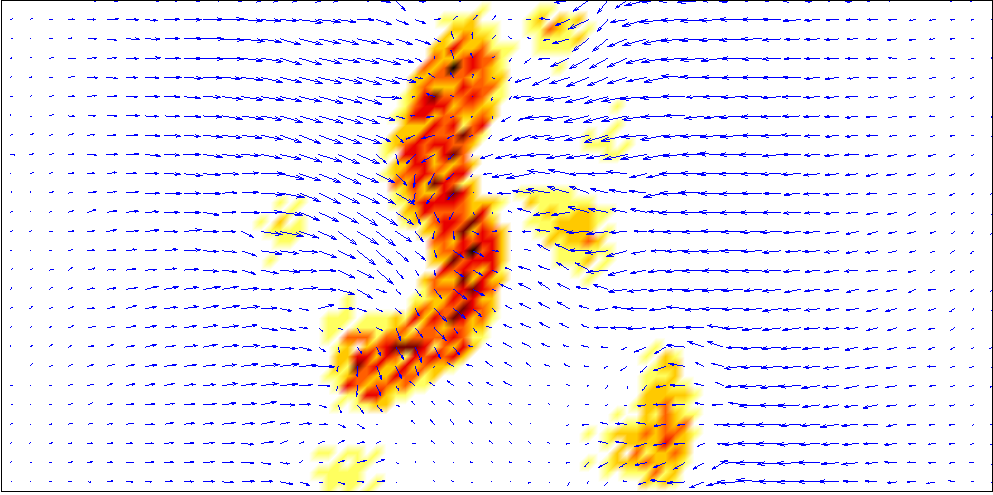

Supplement: S1 Fig — (TIFF) [file pone.0191089.s001.tiff]
