# Supplementary material for: Cell death as a trigger for morphogenesis
Source: PLoS One. 2018 Mar 22;13(3):e0191089. doi: 10.1371/journal.pone.0191089 (PMC5863959; doi:10.1371/journal.pone.0191089)
Supplement: S1 Table — (PDF) [file pone.0191089.s002.pdf]

Parameter	Symbol	Values
Simulation time step	Δt	2s
Spring constant	K	2.2e-8
Coefficient of friction ratio	ζ	0.4e-6
Bond rigidity	s_b , s_{ba}	0.08
Cell radius	R_i	4.125 μm
EPS shoving scale	α	2.5
Diffusion coefficient of cells	D_c	0, 0.01 $\mu m^2 s^{-1}$
Threshold for cell-cell bond creation	δ_c	$1.0\ (\alpha R_i + \alpha R_j)$
Threshold for cell-cell bond breaking	δ_d	$1.4\ (\alpha R_i + \alpha R_j)$
Threshold for cell-agar surface bond creation	δ_{ca}	$1.0\ \alpha R_i$
Threshold for cell-agar surface bond breaking	δ_{da}	$1.8\ \alpha R_i$
Volumetric cell density	Φ	0.16
Dimensions of the system	L_x, L_y, L_z	90, 1440, 360 μm
Width of cell death region	W_d	1000 μm
Height of cell death region	H_d	36 μm

Table S1: Default parameters, constants, and expressions used in the simulations.
