# Supplementary material for: Cell death as a trigger for morphogenesis
Source: PLoS One. 2018 Mar 22;13(3):e0191089. doi: 10.1371/journal.pone.0191089 (PMC5863959; doi:10.1371/journal.pone.0191089)
Supplement: S1 Text — (PDF) [file pone.0191089.s003.pdf]

Supplementary Information for “Cell Death as a Trigger for Morphogenesis”

Boris Aguilar, Ahmadreza Ghaffarizadeh, Christopher D. Johnson, Gregory J. Podgorski, Ilya Shmulevich, and Nicholas S. Flann

Parameter	Symbol	Values
Simulation time step	Δt	2s
Spring constant	K	2.2e-8
Coefficient of friction ratio	ζ	0.4e-6
Bond rigidity	s_b , s_{ba}	0.08
Cell radius	R_i	4.125 μm
EPS shoving scale	α	2.5
Diffusion coefficient of cells	D_c	0, 0.01 $\mu m^2 s^{-1}$
Threshold for cell-cell bond creation	δ_c	$1.0\ (\alpha R_i + \alpha R_j)$
Threshold for cell-cell bond breaking	δ_d	$1.4\ (\alpha R_i + \alpha R_j)$
Threshold for cell-agar surface bond creation	δ_{ca}	$1.0\ \alpha R_i$
Threshold for cell-agar surface bond breaking	δ_{da}	$1.8\ \alpha R_i$
Volumetric cell density	Φ	0.16
Dimensions of the system	L_x, L_y, L_z	90, 1440, 360 μm
Width of cell death region	W_d	1000 μm
Height of cell death region	H_d	36 μm

Table S1: Default parameters, constants, and expressions used in the simulations.

Computation of convergence

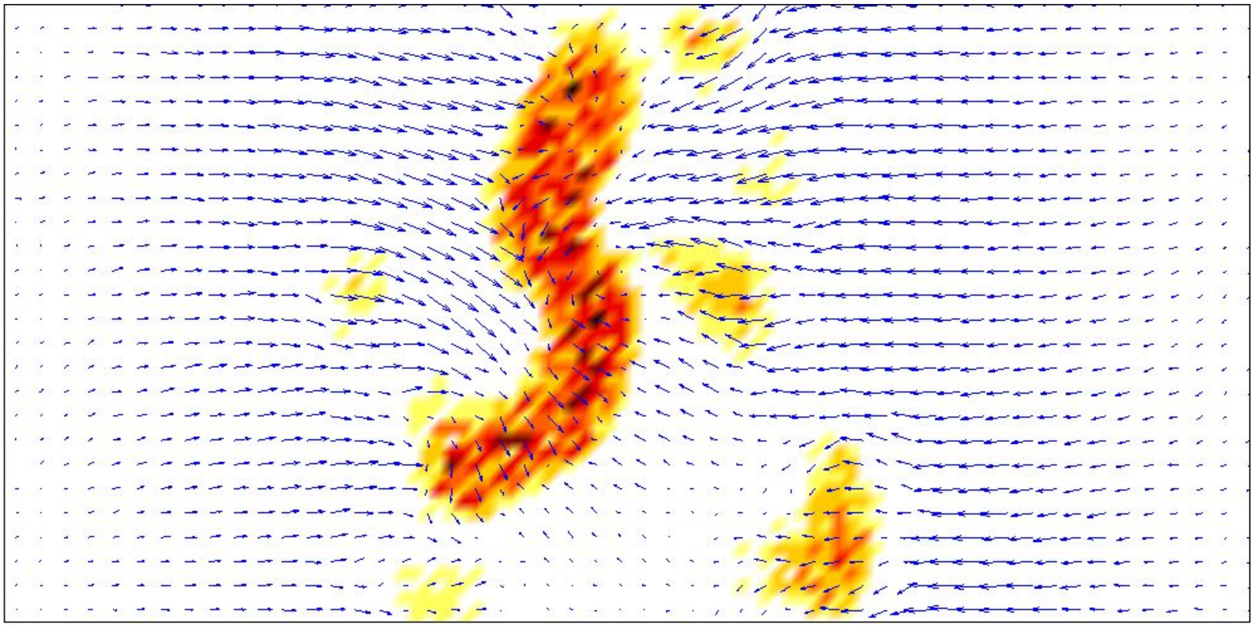

Fig S1. Vector Field and convergence for biofilm surface, demonstrating the aggregate material directional movement.

Initial Configuration of cells and Homogenization process.

We used the program SmolCrowd to generate initial configurations of nonoverlapping cells (spheres). This program requires the volumetric density (Φ), the cell radius, and the dimensions of the system as initial parameters. The configuration obtained from SmolCrowd is then subjected to a relaxation stage for uniformization of local cell density and reduction of high forces that can appear when two cells are in close proximity. The relaxation stage is performed prior to simulations of wrinkle formation due to cell death and before the stress-strain experiments of the “Mechanical stiffness influences wrinkle height and width” section.

The relaxation consists of 2000 simulation steps with periodic boundary conditions in the three directions (no agar), using EPS shoving factor of $\alpha = 1.8$, a weak random term ($D_c = 0.0005$), and $\square_c = \square_d = 1.0(2\alpha R_i)$. These parameters, which in the main text are referenced as “weak forces”, minimize differences in local density of cells (Standard Deviation) in different regions of the system (See Fig S2). The Number density is computed by dividing the systems in regular voxels (grid size = 45.0μm) and counting the number of cells inside the voxels.

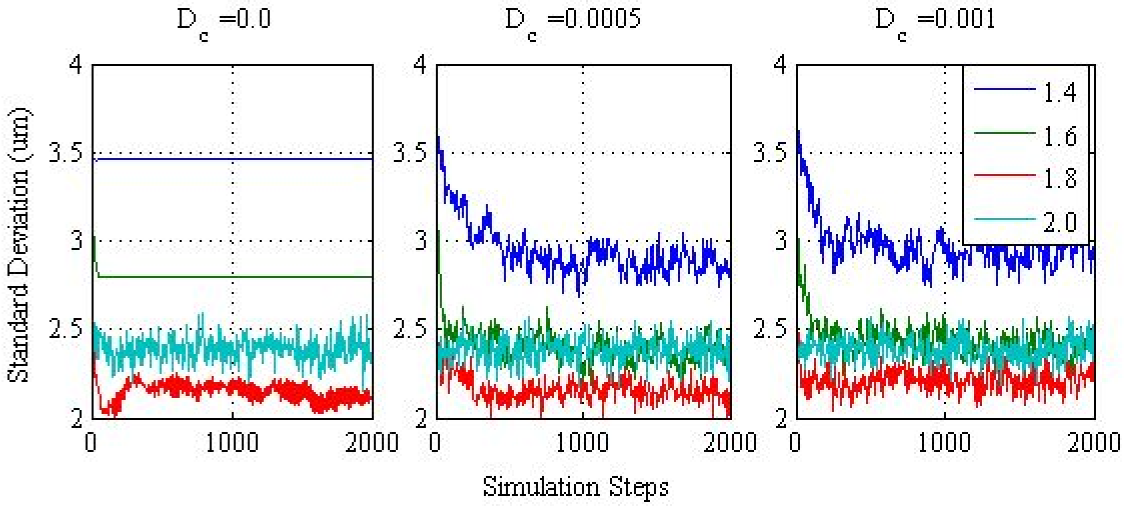

Fig S2. Reduction of standard deviation in local Number density of cells during the relaxation stage. We tested different values of shoving scale (colors) and diffusion coefficients (D_c , $\mu m^2/s$).

This protocol allows to generate configurations with different volumetric densities and dimensions for the wrinkling analysis. Figure S3 shows a comparison of the variability of local density of cells, before and after the relaxation stage, and a snapshot of final configuration of cells for a rectangular systems of $L_x = 180,\ L_y = 900,\ L_z = 360$.

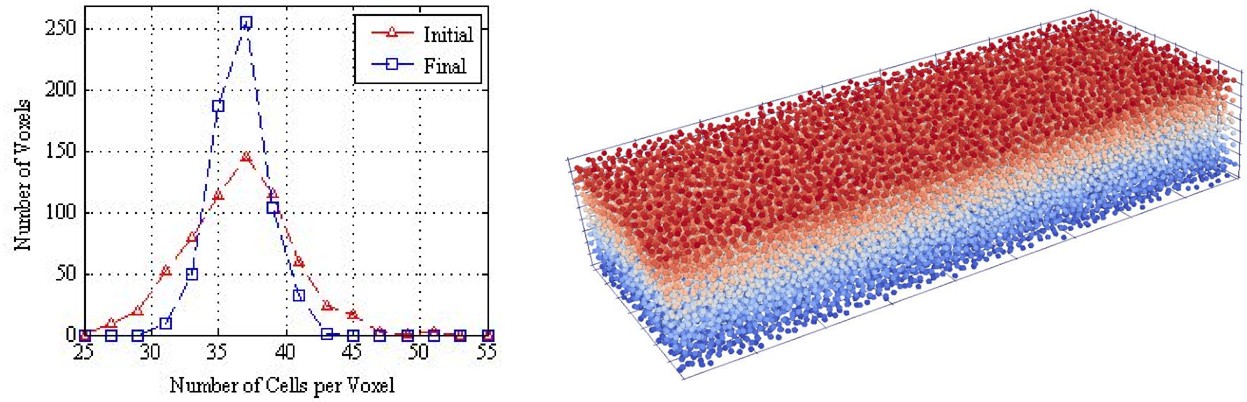

Fig S3. Right: Configuration after the relaxation phase. Color indicates the **x** coordinate of the cells (Height). **Left:** Histograms of number of cells per Voxel from SmolCrowd (Initial) and after Relaxation process (Final). The dimensions of the systems are $L_x = 180,\ L_y = 900,\ L_z = 360$ μm; the volumetric density is $\Phi = 0.12$; and the Voxels have a size of 45 μm.

Simulation convergence

We run the simulations until the number of moving cells is zero. A cell is classified as a moving cell if the magnitude of its velocity is smaller that a threshold value, v_{max} . We used a threshold value of v_{max} = 0.0001μm/s, and a threshold value of $\Delta t = 2$ for the simulations of wrinkle formation induced by cell death, and v_{max} = 0.01μm/s and $\Delta t = 2$ in the stress-strain experiments. Fig S4 (Left) shows how the number of moving cells decreases with the number of simulations steps, converging to zero. Similarly, Fig S4 (Right) shows that the height of the wrinkle reaches a stable value when the system reaches convergence. We tried two time steps

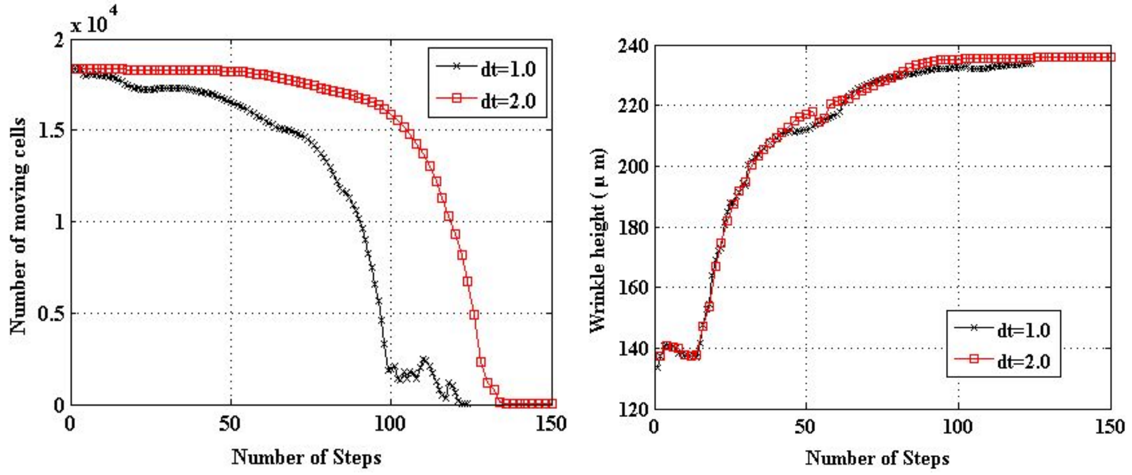

Figure S4. Left: Number of moving cells versus the number of simulation steps. **Right:** Wrinkle height versus the number of steps. The dimensions of the systems are $L_x = 180,\ L_y = 900,\ L_z = 360\ \mu$m; the volumetric density is $\Phi = 0.12$, and $s_b = 0.04$. A single wrinkle is induced by cell death in the center with W_D = 600 and H_D = 36.

Progressive cell death

The spherical agents that are marked as dying cells reduce their volume (V) according to:

$$\frac{dV}{dt} = -\gamma V$$

Cells are removed when the cell radius falls below 0.5 micrometers. Figure S5 shows how the wrinkle height changes with the width of CDP.

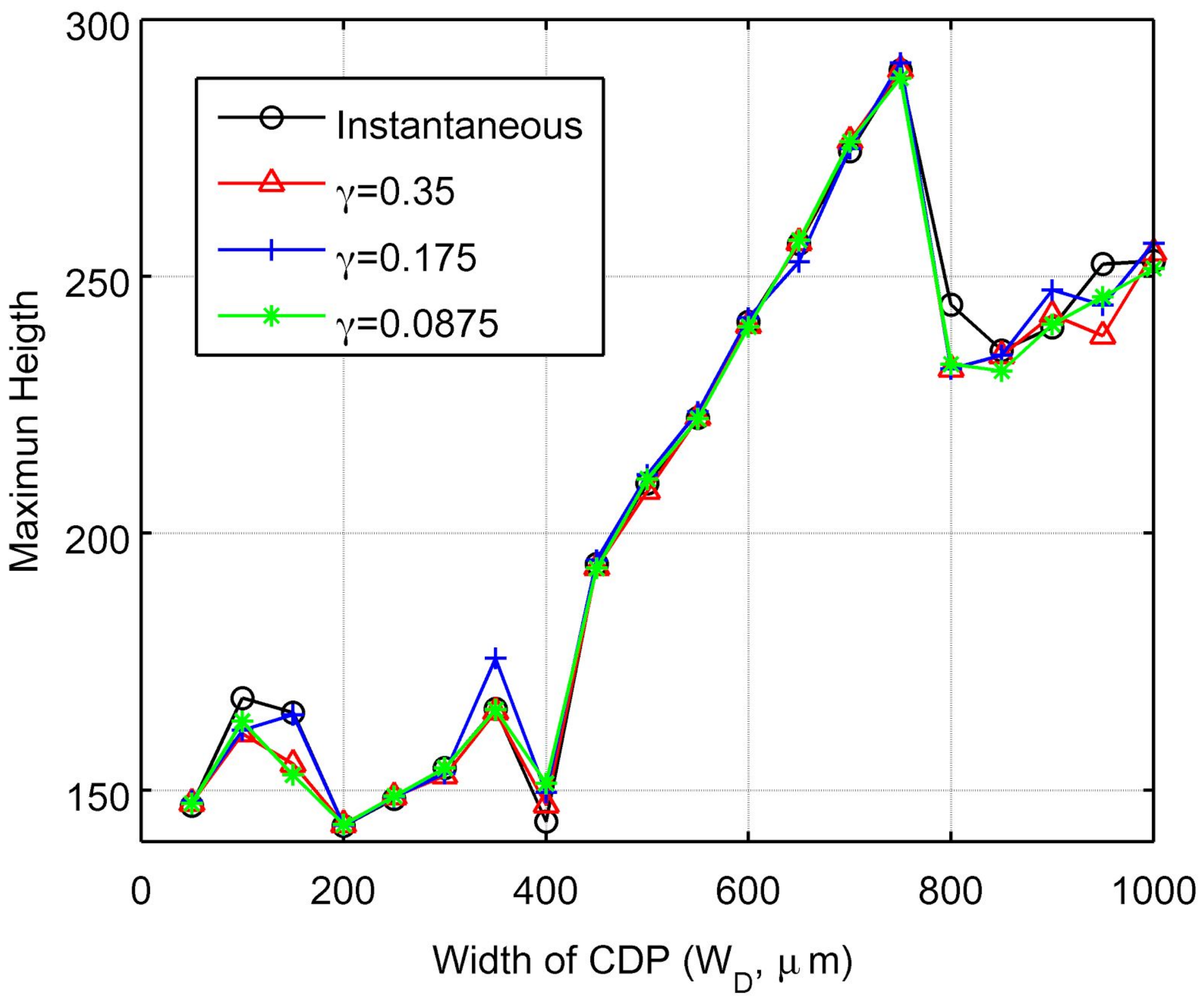

Fig. S5. Colony height (micrometers) versus the width of cell death region (W_D) for various values of γ, the volume shrinking rate. The dimensions of the systems are $L_x = 180,\ L_y = 900,\ L_z = 360\ \mu$m; the volumetric density is $\Phi = 0.16$, $s_b = 0.08$, H_D = 36.
